# Clinical characteristics of genital chlamydia infection in pelvic inflammatory disease

**DOI:** 10.1186/s12905-016-0356-9

**Published:** 2017-01-13

**Authors:** Sung Taek Park, Suk Woo Lee, Min Jeong Kim, Young Mo Kang, Hye Min Moon, Chae Chun Rhim

**Affiliations:** 1 Department of Obstetrics and Gynecology, Hallym University Medical Center, 22 Gwanpyeong-ro 170beon-gil, Dongan-gu, Anyang-si, Gyeonggi-do 431-796 South Korea; 2 Department of Obstetrics and Gynecology, College of Medicine, The Catholic University of Korea, 222, Banpo-daero Seocho-gu, Seoul, 06591 South Korea

**Keywords:** CA-125, Chlamydia infection, Pelvic Inflammatory Disease

## Abstract

**Background:**

Chlamydia infection in acute pelvic inflammatory disease (PID) is associated with serious complications including ectopic pregnancy, tubal infertility, Fitz-Hugh-Curtis syndrome and tubo-ovarian abscess (TOA). This study compared clinical and laboratory data between PID with and without chlamydia infection.

**Methods:**

The medical records of 497 women who were admitted with PID between 2002 and 2011 were reviewed. The patients were divided into two groups (PID with and without chlamydia infection), which were compared in terms of the patients’ characteristics, clinical presentation, and laboratory findings, including inflammatory markers.

**Results:**

The chlamydia and non-chlamydia groups comprised 175 and 322 women, respectively. The patients in the chlamydia group were younger and had a higher rate of TOA, a longer mean hospital stay, and had undergone more surgeries than the patients in the non- chlamydia group. The erythrocyte sedimentation rate (ESR), C-reactive protein (CRP), and CA-125 level were higher in the chlamydia group than in the non-chlamydia group, but there was no significant difference in the white blood cell count between the two groups. The CA-125 level was the strongest predictor of chlamydia infection, followed by the ESR and CRP level. The area under the receiving operating curve for CA-125, ESR, and CRP was 0.804, 0.755, and 0.663, respectively.

**Conclusions:**

Chlamydia infection in acute PID is associated with increased level of inflammatory markers, such as CA-125, ESR and CRP, incidence of TOA, operation risk, and longer hospitalization.

## Background

Pelvic inflammatory disease (PID) is caused by colonization of the endocervix by microorganisms, which then ascend to the endometrium and fallopian tube. Inflammation can be at any point along a continuum that includes endometritis, salpingitis, and peritonitis [1]. PID, one of the most important infections in sexually active women of reproductive age, is a major public health problem [2].

The polymicrobial etiology of PID can be delineated artificially into sexually transmitted infections that colonize the upper genital tract, including *Chlamydia trachomatis, Neisseria gonorrhoeae*, and endogenous microorganisms found in the vagina, particularly anaerobic bacteria such as *Gardnerella vaginalis, Haemophilus influenzae,* enteric Gram-negative rods, and *Streptococcus agalactiae* [3, 4].

*Chlamydia trachomatis* is a Gram-negative bacterium that infects the columnar epithelium of the cervix, urethra, and rectum and a common bacterial cause of sexually transmitted infections [5]. Chlamydia infection is associated with a wide spectrum of upper genital tract pathologies, ranging from asymptomatic endometritis to symptomatic salpingitis, peritonitis, tubo-ovarian abscess (TOA), Fitz-Hugh-Curtis syndrome (FHCS) characterized by inflammation in perihepatic capsules, and long-term sequelae such as infertility, ectopic pregnancy, and chronic pelvic pain [6–8]. Moreover, chlamydia infection can lead to obstetric complications, including premature rupture of the membrane, chorioamnionitis, premature delivery, puerperal and neonatal infections, and an increased risk of the development of cervical carcinoma [9]. Prompt treatment and screening for chlamydia infections in women of reproductive age is essential to prevent severe damage to the reproductive organs; Consequently, chlamydia screening and treatment programs have been implemented in many countries [10].

A few studies on the comparison between chlamydia and non-chlamydia infection in women with PID have been reported; however, previous studies mainly focused on the comparisons of clinical characteristics and sequelae [11–13].

This study compared the clinical course of PID with and without chlamydia infection by examining patients’ clinical and laboratory data on admission.

## Methods

### Participants

We reviewed the medical records of 1,422 women diagnosed with PID, salpingitis, endometritis, FHCS, or a TOA, who were treated at St. Vincent’s Hospital (Suwon, Korea) between January 2002 and December 2011 with a diagnosis at discharge of PID, salpingitis, endometritis, or TOA. This retrospective study was approved by the Institutional Review Board of Catholic University of Korea(C11RIMI0130V).

The definition of PID was based on a clinical history of abdominal pain and on clinical findings of abdominal pain, cervical motion tenderness, and adnexal tenderness. In addition, at least one or more minor criterion was required: temperature ≥38.3°C, an abnormal cervical or vaginal mucopurulent discharge, an elevated erythrocyte sedimentation rate (ESR) or C-reactive protein (CRP) level, and laboratory documentation of a cervical infection with *Neisseria gonorrhoeae* or *Chlamydia trachomatis* [14]. All patients underwent pelvic ultrasonography or abdominal and pelvic computed tomography (CT).

FHCS was indicated the following: (1) abdominal CT showing pelvic inflammation with contrast enhancement of hepatic capsules, or (2) adhesions between the liver and the diaphragm or the liver and the anterior abdominal wall as detected by laparoscopic surgery [15]. TOA was diagnosed based on the presence of an abscess, indicated by a tender adnexal mass or masses and ultrasonographic or abdominal CT findings supporting an abscess. The sonographic diagnosis of TOA was based on the demonstration of a complex, cystic mass with thick, irregular walls, partitions, internal echoes, and absent peristalsis [16]. The finding of TOA by CT was based on adnexal wall thickening and enhancement with complex fluid collections that may contain internal septa and a fluid-debris level [17]. The diagnosis of TOA was based on satisfying the PID criteria and the presence of at least one complex pelvic mass as mentioned above for ultrasonographic or CT findings [14].

Participants were excluded based on the following criteria: (1) no assessment of the CA-125 level (*n* = 378); (2) not performing vaginal swab for a chlamydia infection (*n* = 323); (3) not meeting the diagnostic criteria of PID (*n* = 167); (4) the presence of a uterine or adnexal pathology, including epithelial and germ cell ovarian neoplasms, endometriosis, leiomyoma, or adenomyosis (*n* = 30); (5) antibiotic use over the preceding 7 days (*n* = 21); and (6) obstetric delivery or abortion in the previous 30 days (*n* = 6). We measured the axillary body temperature of all patients, with fever defined as a temperature of ≥38°C. The white blood cell (WBC) count for each patient was quantified using the XE-2100™ automated hematology system (Sysmex Inc., Mundelein, IL, USA). The intra- and inter-assay coefficients of variation (CVs) were 1.7 and 1.9%, respectively. Leukocytosis was defined as a WBC count greater than ≥11,000/mm^3^. The serum ESR was measured using a modified version of the Westergren method with a Test-1 automated analyzer (Ailfax, Padova, Italy). The intra- and inter-assay CVs were 3.5 and 3.4%, respectively. The upper limit of normal for the ESR in females ≤50 years of age is 20 mm/h [18]. The serum CRP level was measured by a turbidimetric immunoassay (TIA) using a Hitachi 7600-110® automatic analyzer (Hitachi Co., Tokyo, Japan). The intra- and inter-assay CVs were 5.4 and 2.7%, respectively. Normal serum CRP levels range from 0 to 0.6 mg/dl. The serum CA-125 level was measured by a chemiluminescent microparticle immunoassay (CMIA) using a CA-125 II™ kit (Abbott Architect, Inc., Chicago, IL, USA). The intra- and inter-assay CVs were 2.4 and 3.9%, respectively. CA-125 was considered abnormal if the concentration was ≥35 IU/ml. Endocervical swabbing and testing by real-time polymerase chain reaction (PCR) were done to identify *Neisseria gonorrhoeae* and *Chlamydia trachomatis*.

The two groups were compared statistically using a two-tailed Student’s *t*-test and *χ* ^2^ test. Logistic regression analysis was performed to determine the relationship between chlamydia infection in PID and inflammatory markers. Since CA-125 is influenced by age, smoking and pelvic pathology, the association between CA-125 and chlamydia infection in PID was evaluated by logistic regression analysis and analysis of covariance (ANCOVA). The covariates were age, parity, histories of pelvic surgery or cesarean section, use of intrauterine device (IUD), menstrual problems, alcohol consumption, smoking, and TOA. A receiver operating characteristic (ROC) curve analysis was used to determine the relationship between chlamydia infection in PID and inflammatory markers. *p* < 0.05 was considered to indicate statistical significance.

## Results

Among the 497 females diagnosed with acute PID, 175 (35.2%) and 322 (64.8%) were in the chlamydia and non-chlamydia groups, respectively. The patients in the chlamydia group were younger than those in the non-chlamydia group, and the patients in the chlamydia group were less likely to be married and to have had children than those in the non-chlamydia group. There were no significant differences between the groups regarding abortion, previous pelvic surgery, previous PID episodes, IUD insertion, barrier contraceptive use, incidence of menstrual problems, and alcoholic and smoking history. The patients in the chlamydia group had more symptom of right upper quadrant pain and the patients in non-chlamydia group had more symptom of lower abdominal pain (Table 1).

**Table 1:** Demographic data of patients with PID

	PID with chlamydia (*N* = 175)	PID without chlamydia (*N* = 322)	*P*
Mean age (years)	27.9 ± 8.2	32.4 ± 8.3	<0.0001
Married (%)	71 (40.6)	223 (69.3)	<0.0001
Parity	0.7 ± 0.9	1.2 ± 1.0	<0.0001
Abortion	1.0 ± 1.2	1.2 ± 1.5	0.36
Cesarean sections (%)	19 (10.9)	89 (27.6)	<0.0001
Previous pelvic surgery (%)	35 (20.0)	65 (20.2)	0.530
History of PID	26 (14.9)	61 (18.9)	0.269
Use of IUD (%)	18 (10.3)	38 (11.8)	0.658
Barrier contraceptive use (%)	22 (12.6)	46 (14.3)	0.682
Main symptoms (%)			
Low abdominal pain	110 (62.9)	276 (85.7)	0.001
Acute abdomen	19 (10.9)	19 (5.9)	0.321
Vaginal discharge	5 (2.9)	6 (1.9)	0.320
Right upper quadrant pain	34 (19.4)	5 (1.6)	<0.0001
Febril sensation	7 (4.0)	16 (5.0)	0.628
Menstrual problem			
Menorrhagia (%)	20 (14.0)	15 (9.2)	0.215
Dysmenorrhea (%)	20 (14.0)	15 (9.2)	0.215
Alcohol (%)	72 (41.1)	107 (33.2)	0.096
Smoking (%)	45 (25.7)	74 (22.3)	0.510

*PID* pelvic inflammatory disease, *IUD* intrauterine device

There were no significant differences in the mean WBC count, leukocytosis, mean temperature, and the incidence of fever between the two groups, while the ESR, CRP, and CA-125 level were higher in the chlamydia group than the in non-chlamydia group (all *P* < 0.001; Table 2).

**Table 2:** Clinical and laboratory data in patients with PID

	PID with chlamydia (*N* = 175)	PID without chlamydia (*N* = 322)	*P*
Mean temperature (°C)	37.2 ± 0.8	37.2 ± 0.9	0.893
Fever (%)	36 (20.6)	73 (22.7)	0.650
Mean WBC count x10^3^/μl	10.3 ± 4.0	10.2 ± 4.5	0.948
Leuocytosis (%)	63 (36.0)	102 (31.7)	0.836
ESR (mm/h)	45.0 ± 26.6	23.5 ± 24.5	<0.0001
CRP (mg/dl)	7.6 ± 7.0	4.8 ± 6.5	<0.0001
CA 125 (U/mL)	130.7 ± 174.6	23.8 ± 40.0	<0.0001
Abnormal CA 125 (%, > 35 U/mL)	115 (65.7)	42 (13.0)	<0.0001
Other sexually transmitted infection (%)			
Neisseria gonorrhoeae	11(6.3)	1(0.3)	<0.0001
Mycoplasma genitalium	13(7.4)	4(1.2)	0.001
Mycoplasma hominis	9 (5.1)	11(3.4)	0.349
Ureaplasma urealyticum	19(10.9)	17(5.3)	0.029
Trichomonas vaginalis	14(8.0)	16(5.0)	0.236
Hospital day (days)	8.2 ± 2.7	7.2 ± 2.4	0.016
Recurrent PID (%)	9 (5.1)	5 (1.6)	0.051
TOA (%)	45 (25.7)	24 (7.4)	<0.0001
FHC syndrome (%)	69 (39.4)	2 (0.6)	<0.0001
Operation (%)	39 (22.2)	32 (9.9)	<0.0001

*PID* pelvic inflammatory disease, *WBC* White blood cell, *ESR* erythrocyte sedimentation rate, *CRP* C-reactive protein, *TOA* tubo-ovarian abscess, *FHC* syndrome Fitz-Hugh-Curtis syndrome

**Fig. 1:**
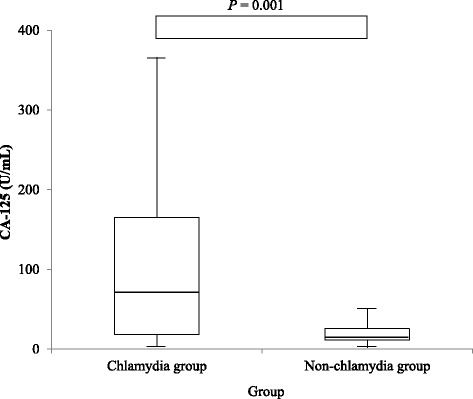
Mean CA-125 level in chlamydia and non-chlamydia group. After adjustment for age, smoking, and the incidence of TOA, a difference of CA-125 level between two groups showed a statistical significance (*P* = 0.001). *P* value was calculated by analysis of covariance

Logistic regression analysis was used to identify the independent variables that affected the serum CA-125 level. Age, smoking, and TOA were independent predictors of the serum CA-125 level (*P* = 0.003, 0.013, and < 0.001, respectively; Table 3). After adjusting for age, smoking, and the incidence of TOA, a significant difference in CA-125 level remained between the chlamydia and non- chlamydia groups (*P* = 0.001; Fig. 1).

**Table 3:** Logistic regression analysis of independent variables that affect the serum CA-125 level between chlamydia and non-chlamydia group in acute PID

Variable	Odds ratio (95% confidence interval)	*P*
Age	-3.00 (-4.073 ~ -0.852)	0.003
Smoking	2.53 (4.032 ~ 20.174)	0.013
TOA	10.84 (129.819 ~ 187.289)	<0.0001

The mean length of hospital stay differed significantly; it was 8.2 ± 2.7 days for the chlamydia group and 7.2 ± 2.4 days for the non-chlamydia group (*P* = 0.001). In the non- chlamydia group, two cases (0.6%) were diagnosed with FHCS, versus 39.4% of the patients in the chlamydia group (*P* < 0.001). The incidence of surgery was higher in the chlamydia group (Table 2; 22.2 vs. 9.9%, respectively; *P* < 0.001). Although the rate of recurrent PID was higher in the chlamydia group, the difference between the two groups was not significant (*P* = 0.051). TOA was diagnosed in 13.9% of the patients hospitalized for PID (69 of 497). The incidence of TOA was higher in the chlamydia group than in the non-chlamydia group (Table 2; 25.7 vs. 7.4%, respectively; *P* < 0.001). In the non-chlamydia group, the occurrence of TOA was associated with IUD insertion, except in one case.

**Fig. 2:**
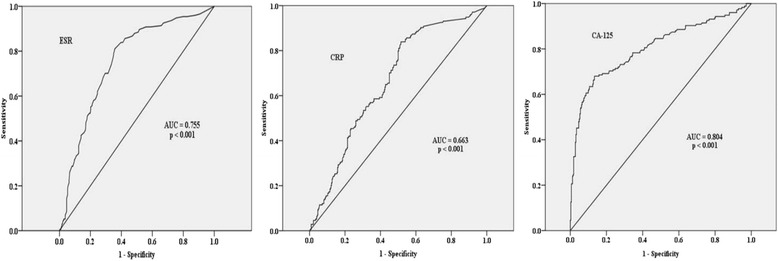
ROC curve of erythrocyte sedimentation rate (ESR), C-reactive protein (CRP) and CA 125 in the diagnosis of chlamydia infection of PID

A ROC curve was constructed and used to select cut-off values as predictors of chlamydia infection. The strongest predictor of chlamydia infection was CA-125, followed by ESR and CRP. The area under the receiving operating curve (AUC) for CA-125, ESR, and CRP was 0.804, 0.755 and 0.663, respectively (Fig. 2). The cut-off values for ESR, CRP, and CA-125 are shown in Table 4; a cut-off value for the leukocyte count was not determined.

**Table 4:** Diagnostic values of inflammatory markers in the diagnosis of chlamydia infection

	Cut-off value	Sensitivity	Specificity	Area under the curve	*P*
ESR (mm/h)	19.5	84%	60%	0.755	<0.001
CRP (mg/dl)	3.6	60%	60%	0.663	<0.001
CA 125 (U/mL)	34.5	66%	87%	0.804	<0.001

## Discussion

Chlamydia infection in the lower genital tract is common in reproductive-age women, affecting mainly women younger than 25 years and 20–30% of PID cases have been attributed to *Chlamydia trachomatis* [2]. Studies have proved that chlamydia infection is associated with an increased risk of PID and longer PID hospitalization [19, 20]. Two-thirds of all cases of tubal factor infertility and one-third of all cases of ectopic pregnancy might be due to chlamydia infection [21].

The mechanism of PID in chlamydia infection is not yet known. Chlamydial heat shock protein 60 expression induced by the cell-mediated immune response drives inflammatory responses associated with severe sequelae of the female reproductive system [22]. A study using nonhuman primate models of lower genital tract chlamydia infection showed chronic salpingitis with extensive tubal scarring, distal tubal obstruction, and peritubal adhesions caused by an ascending infection due to cervical inoculation with chlamydia [23].

TOA is one of the most severe complications of PID and can lead to significant morbidity and occasionally mortality. TOA is caused by an infection ascending to the fallopian tube involving aerobes and anaerobes, and tubal blockage due to tubular endothelial damage and infundibular edema, ovarian invasion by organism through the site of ovulation, and adhesion formation between the ovary and fallopian tube resulting in the development of necrosis inside this complex mass, anaerobic growth, and abscess cavities [24]. An animal study showed that chlamydia infection in the lower genital tract facilitates the formation of abscesses by aerobic and anaerobic bacteria synergistically [25]. We showed that chlamydia infection was associated with a higher prevalence of TOA and an increased risk of surgery in our study.

The diagnosis of PID is based on clinical criteria. However, given the nonspecific nature of the clinical diagnosis, several diagnostic tools, including laboratory tests (e.g., WBC count, ESR, CRP level, and CA-125 level), as well as imaging, endometrial biopsy, and laparoscopy, have been introduced.

CA-125 is a glycoprotein found in the blood that is commonly used as a tumor marker of neoplasia because it can indicate ovarian cancer. CA-125 has been demonstrated in the peritoneum and epithelial tissues of Müllerian origin in the female genital tract, such as the endometrium and fallopian tube [26, 27]. Peritoneal irritation such as endometriosis, salpingitis, ruptured ectopic pregnancy, and pelvic surgery often markedly elevates the level of circulating CA-125, so the serum CA-125 level has been recommended as a useful test for acute PID [28]. Several studies have found correlations between the CA-125 level and PID [29, 30]. However, no study has examined the difference in the CA-125 level in patients with PID according to the presence of a chlamydia infection. In our study, we found a significant difference in the CA-125 level between the chlamydia and non-chlamydia groups.

The increase in serum CA-125 in patients with PID and a chlamydia infection can be explained in several ways. First, increased CA-125 levels in endometriosis might be associated with peritoneal leakage of an endometriotic cyst and an inflammatory reaction in the mesothelial cells of the peritoneum [31]. We believe that chlamydia infections cause more peritoneal irritation than other genital pathogens. Second, CA-125 antigen is found in normal adult fallopian tube epithelium, suggesting that severe inflammation of the fallopian tube can increase the serum CA-125 concentration and it is a useful marker for the clinical diagnosis of salpingitis [29]. It is possible that a pelvic chlamydia infection causes more severe inflammation of the fallopian tube than other pelvic infections. Third, TOA is a major complication of acute PID that is associated with increased serum CA-125 levels in the range of neoplastic activity [32].

To our knowledge, our study is among the first to compare clinical and inflammatory parameters between chlamydia and non-chlamydia infection in acute PID. However, our study had several limitations. First, as a retrospective study, many participants were excluded from our study because all patients with PID are not routinely examined for both CA-125 and vaginal swab for chlamydia. This might lead to selection bias. Second, the diagnosis of PID in our study was based on the medical history, clinical findings, and elevation of inflammatory markers using the CDC criteria. We did not perform a laparoscopy or endometrial biopsy to confirm PID and we failed to confirm the chlamydia infection in the pelvic organs. Although the clinical symptoms of PID in combination with one or two inflammatory parameters can increase the specificity of the diagnosis of PID, laparoscopy is recommended for confirming the diagnosis or if there is no improvement within 72 h despite adequate antibiotic therapy [33].

## Conclusion

In our study, we found that genital chlamydia infection in acute PID was associated with increased inflammatory markers such as CA-125 and ESR, higher incidence of TOA and operation risk, and longer hospitalization. We need to answer other questions. Do PID and TOA have different mechanisms in genital chlamydia infection? Is there a correlation between the CA-125 level and severity of PID? Can we predict TOA or other severe sequelae using the level of CA-125 or other inflammatory markers? To answer these questions, additional experimental studies are needed to determine the mechanism of pelvic inflammation and related complications caused by a genital chlamydia infection, and to elucidate the association between the changes in inflammatory markers and the severity of infection.

## Abbreviations

CMIA: Chemiluminescent microparticle immunoassay
CT: Computed tomography
CVs: Coefficients of variation
FHCS: Fitz-Hugh-Curtis syndrome
IUD: Intrauterine Device
PCR: Polymerase chain reaction
PID: Pelvic inflammatory disease
TIA: Turbidimetric immunoassay
TOA: Tubo-ovarian abscess
WBC: The white blood cell
